# Single-cell transcriptomics reveals keratinocyte dynamic processes associated with S100a4 expression in psoriasiform dermatitis

**DOI:** 10.3389/fimmu.2025.1744860

**Published:** 2026-01-23

**Authors:** Huiqin Wang, Yuan Ding, Shirong Yu, Tingting Li, Dezhi Zhang, Zhenqun Weng, Caying Wu, Zhenrui Wang, Yiqi Wang, Yaqin Ma, Weidong Wu

**Affiliations:** 1 Department of Dermatology and Venereology, People’s Hospital of Xinjiang Uygur Autonomous Region, Urumqi, China; 2 Xinjiang Clinical Research Center for Dermatology and Venereology, Urumqi, China; 3 Xinjiang Key Laboratory of Dermatology Research, Urumqi, China; 4 Teaching Department, People’s Hospital of Xinjiang Uygur Autonomous Region, Urumqi, China

**Keywords:** immune regulation, keratinocytes, psoriasis, S100a4 knockout, single-cell RNA sequencing

## Abstract

**Background:**

Psoriasis is a common autoimmune skin disease with high morbidity and associated complications, characterized by epidermal hyperplasia and cutaneous infiltration of immune cells. The role of S100A4, a key antimicrobial peptide, is highly expressed in psoriatic skin and has aroused considerable interest in recent years, yet its specific function and associated molecular mechanisms remain elusive.

**Methods:**

Using CRISPR/Cas9 to generate S100a4 gene knockout mice, imiquimod was continuously applied to the back skin to induce the psoriasis disease model. Single-cell RNA sequencing (scRNA-seq) was employed to investigate changes in epidermal cell composition and gene expression profiles in mice subjected to different treatments. Multiple bioinformatics analyses were conducted to elucidate the biological role of S100A4 in psoriasis pathogenesis.

**Results:**

We observed that S100a4 knockout mice exhibited significant pathological improvement in psoriasis-like lesions, including reduced inflammatory cell infiltration and decreased epidermal hyperplasia. The results of scRNA-seq revealed that after S100a4 knockout, the pathologically relevant keratinocyte subpopulation was significantly reduced, and the related tumor necrosis factor (TNF) and interleukin-17 (IL-17) signal transducers and activator of keratinization were downregulated. Moreover, S100a4 depletion moderated the abnormal proliferation and differentiation dynamics of keratinocytes. Additionally, Klf9-mediated transcriptional dysregulation of Krt15 in keratinocytes was identified as a key driver of hyperkeratosis, while S100a4 deficiency contributed to restoring cellular homeostasis in this process.

**Conclusions:**

Our findings suggest a potential pathogenic role for S100A4 in psoriasis and highlight previously uncharacterized cell-specific transcriptional landscapes and regulatory mechanisms. Our results provide novel insights into the complex pathology of psoriasis and could offer important clues for the development of new targeted therapeutic strategies.

## Introduction

Psoriasis is a chronic immune-mediated inflammatory skin disease, affected by heredity and various environmental risk factors, mainly manifested as scaly erythematous spots or plaques, commonly found on the scalp, elbows, knees, waist, and lower back. In severe cases, the lesions could be generalized all over the body and even involve the nails and joints, resulting in the formation of psoriatic arthritis. Furthermore, psoriasis might be comorbid with other systemic diseases, such as cardiovascular diseases, metabolic syndrome, and mental health disorders, which seriously undermines the quality of life of patients (1–3). Psoriasis is marked by sustained inflammation leading to uncontrolled skin keratinocyte (KC) proliferation and dysfunctional differentiation, and disturbances in the innate and adaptive immune response are key contributors to the development and sustainment of inflammation in psoriasis; for instance, activation of the tumor necrosis factor-α (TNF-α)/interleukin-23 (IL-23)/IL-17 axis among immune cells is considered to be a central driver of psoriasis pathogenesis (4–6). Over the decades, great strides have been made in the pathogenic mechanisms of psoriasis, with increasing evidence that various cytokines and interactions between different cell types constitute an intricate cascade of regulation that ultimately leads to the onset and progression of psoriasis (7–9). Many genes involved in predisposition to psoriasis have been found and tend to cluster to a small number of immune pathways (10, 11); however, not all the underlying genes have been conclusively identified, so that further exploration of disease-important genes and their functions and mechanisms is still required.

The S100 proteins are a group of indispensable antimicrobial peptides (AMPs) in psoriasis and participate in a wide spectrum of cellular processes, particularly in KCs where their expression and secretion lead to the production of several pro-inflammatory cytokines that contribute to autoimmune system activation and psoriasis pathogenesis (12). Although many S100 proteins are highly expressed in psoriatic skin, S100A4 stands out due to its unique expression pattern and multifunctional signaling profile. Studies have shown that S100A4 is predominantly upregulated in the dermis of psoriatic skin, in contrast to other S100 proteins such as S100A7 and S100A8/9, which are mainly expressed in the epidermis (13, 14). Moreover, S100A4 is primarily recognized for its pro-inflammatory role and considered one of the most decisive mediators in disease progression (15). As an important member of the S100 family, S100A4 has both intra- and extracellular functions. Intracellularly, it influences cell metastasis, apoptosis, and stemness through binding to multiple target proteins, including the tumor suppressor p53 and proteins involved in cytoskeletal rearrangement and cell motility (16, 17). When released extracellularly, it acts as a damage-associated molecular pattern (DAMP) that could activate downstream signaling cascades via interacting with several cell surface receptors, such as the receptor for advanced glycation end products (RAGE) and toll-like receptor 4 (TLR4), triggering processes like inflammation and tissue fibrosis (18, 19). Moreover, in the extracellular space, S100A4 also attracts immune cells, particularly enhancing the recruitment and chemotaxis of numerous inflammatory cells, thereby modulating inflammation and immune function (20, 21). It has been extensively reported that S100A4 is associated with a variety of disease activities including autoimmune disease, and that S100A4 regulation of immune cell function has a profound role in promoting the pathogenesis of pro-inflammatory conditions (22). Targeting S100A4 therapeutically has been shown to effectively reduce dermal thickening in diseases like systemic sclerosis, prevent skin fibrosis, and attenuate inflammatory progression, highlighting its promising potential for targeted therapy (23). Irrespective of these findings, the role of S100A4 in psoriasis remains largely unknown and warrants further investigation.

In recent years, single-cell RNA sequencing (scRNA-seq) has provided an efficient way to analyze specific cell populations in psoriasis at the single-cell level based on differential transcriptomic patterns (24, 25). For example, Qie et al. used scRNA-seq to reveal the transcriptional landscape and heterogeneity of skin macrophages in Vsir knockout (KO) murine psoriasis (26). Wang et al. uncovered that Chrna5 KO had a significant effect on KC proliferation with scRNA-seq analysis and showed that Chrna5 KO downregulated key Jak/signal transducer and activator of transcription signaling pathway in psoriasis (27). In this work, we integrated multiple mouse models of Sham-operated, pathological wild-type (WT), and pathological global KO S100a4, and then utilized scRNA-seq approaches to query the biological and molecular roles of S100A4 in psoriasis. We found that S100A4 deletion effectively alleviated psoriasis-induced skin lesions, attenuated pathological severity by decreasing inflammatory immune cell aggregation and infiltration, and revealed a central gene regulatory network to maintain the homeostasis of subsequent proliferation and differentiation dynamics in KCs. Collectively, these findings provide unprecedented insights into understanding the pathological impact of S100A4 in psoriasis and may serve as a potential direction for future targeted treatment.

## Materials and methods

### Mice and treatment

All procedures involving experiment animals were performed in accordance with the ARRIVE Guidelines for the Care and Use of Laboratory Animals and approved by the Animal Ethics Committee of Xinjiang Uygur Autonomous Region People’s Hospital.

Under specific pathogen-free (SPF) conditions, 6- to 8-week-old female mice (C57BL/6J background, purchased from Cyagen Biosciences) were raised in a suitable environment with moderate light and free access to food and water. For generating KO mice, we designed two guide RNAs (gRNAs) that targeted S100a4. Briefly, prepared Cas9 mRNA and gRNA were injected into zygotes of C57BL/6J mice by electroporation to obtain F_0_ generation mice. Founder mice were selected based on genotyping polymerase chain reaction (PCR) assay results and mated with WT partners to obtain germline transmission. Western blot was used to determine the expression of the S100a4 protein. At least three animal samples were used for each experimental group, and overall, we generated transgenic mice with conditional S100a4 KO using CRISPR/Cas9 technology. For psoriasis modeling, we applied a 62.5-mg dose of 5% imiquimod (IMQ) cream to the backs of mice for either four or seven consecutive days. Based on a comparison of the outcomes, the 4-day regimen was ultimately selected as it consistently induced more robust and pronounced psoriasiform features. During the experiment, body weight and skin changes were measured every 2 days, and the severity of psoriasis-like lesions was assessed using the PASI score, a scoring system that takes into account erythema, thickness, and desquamation (0–4 points). The following scoring was applied: 0, none; 1, mild; 2, moderate; 3, serious; and 4, very serious. All scoring was performed by the same trained investigator who was blinded to the genotype and treatments. In the end, skin tissues were collected and hematoxylin–eosin (H&E) staining was performed for histological evaluation. Dermal inflammatory cell infiltration was quantified by counting nuclei in standardized high-power fields within the dermal area using ImageJ software, while epidermal thickness was measured based on section and microscopic observations.

### Skin cell isolation and scRNA-seq

The dorsal lesion skins from mice were carefully separated, then washed with pre-chilled phosphate-buffered saline (PBS) to clean the surface and cut into small pieces (<1 mm). The skin tissues were digested with a cocktail of collagenase IV, dispase II, and DNase I in RPMI 1640 medium at 37 °C for 45 min. After digestion, the cell suspensions were resuspended and mixed gently, and cell viability was examined by 0.4% trypan blue staining, and the concentration of live cells was adjusted to the ideal concentration. Cellular suspensions were loaded onto the BD Rhapsody™ Cartridge Kit according to the manufacturer’s guidelines, to facilitate the generation of single-cell magnetic beads within the microwells. For library preparation, we constructed scRNA-seq libraries using the BD Rhapsody™ WTA Amplification Kit and subsequently paired-end sequenced each library on an Illumina Novaseq 6000 platform adhering to the manufacturer’s instructions, and utilizing the BD Rhapsody™ sequencing pipeline, we generated expression matrices for further analysis.

### Raw data processing and quality control

The raw unique molecular identifier (UMI) count matrix was first converted into a Seurat object by the Seurat package (v4.3.0) (28). Cells with UMI counts (<1,000), detected genes (<500), or percentage of mitochondrial genes (≥15%) were then filtered out. Potential doublets were further identified and removed using the DoubletFinder (v2.0.3), and genes expressed in fewer than five cells were also excluded. After removing low-quality cells, we log-normalized the data with the NormalizeData function and selected the top 2,000 highly variable genes in each sample for downstream analysis. Data integration was accomplished through the Harmony (29) algorithm and scaling was conducted using the ScaleData function. Principal component analysis (PCA) was executed using the RunPCA function, and the top 30 principal components were selected for dimensionality reduction and subsequent clustering. The main cell clusters were identified using the FindClusters function with resolution set to 0.4, and then visualized with Uniform Manifold Approximation and Projection (UMAP) plots. To determine the cell type of each cluster, we used the FindMarkers function to detect the marker genes for each cluster, and then annotated the cell types meticulously using the ScType tool (30).

### Differentially expressed gene and functional enrichment analysis

The expression of each gene in a given cluster was compared against that in the rest of the cells using the Wilcoxon rank sum test; *p*-values were adjusted for multiple testing using the Bonferroni correction. For computing differentially expressed genes (DEGs), all genes were probed such that the expression difference on a natural log scale was at least 0.5 and the difference of percentage of detected cells was at least 0.2 and adjusted *p*-value was less than 0.05. Clustering and visualization of gene expression patterns were carried out using the clusterGVis package (v0.1.2, https://github.com/junjunlab/ClusterGVis), and Gene Ontology (GO) analysis, Kyoto Encyclopedia of Genes and Genomes (KEGG) analysis, and Gene Set Enrichment Analysis (GSEA) were performed with the clusterProfiler package (v4.6.2) (31).

### Pseudotime trajectory analysis

Monocle2 (v2.26.0) (32) was used for pseudotime analysis, trajectory construction, and calculation of pseudotime-dependent gene expression. The DDRTree (v0.1.5) method is used for dimension reduction. Cell trajectory was visualized according to cell subtypes and cell states. The Basic Differential Analysis algorithm is used to identify DEGs according to pseudotime function. BEAM (Branched Expression Analysis Modeling) was used to identify genes that are regulated in a branching-dependent manner.

### Transcription factor regulatory network analysis

The transcription factor regulatory network modules (regulons) were identified by the pySCENIC (v0.11.2) (33) workflow using default parameters. Activated TFs were identified in the AUC matrix, and differentially activated TFs were selected using the R package limma (34) based on the fold change (logFC ≥ 0.5 or ≤ −0.5) and false discovery rate (FDR ≤ 0.05). To identify cluster-specific regulons, we used the Regulon Specificity Score (RSS) (35) for evaluation. Networks of the modules with TFs and their target genes were visualized by Cytoscape (v3.9.1) (36).

### Cell–cell communication analysis

Cell–cell interactions based on the expression of known ligand–receptor pairs in different cell types were inferred using CellChat (v2.1.1) (37). To identify potential cell–cell communication networks perturbed or induced in mice skins, we followed the official workflow and used the precompiled protein–protein interactions as *a priori* network information. Then, the communication probability was computed using the computeCommunProb function, and finally, the function netAnalysis_signallingRole was applied on the netP data slot to determine senders and receivers in the network.

### Statistical analysis

All statistical analyses were performed using the R (v4.4.2) software, and data were presented as mean ± standard deviation (SD) unless otherwise stated. Wilcoxon rank sum test or unpaired Student’s *t*-test was used to compare two groups of values, and a *p*-value < 0.05 was considered significant.

## Results

### ScRNA-seq reveals cell-type composition of skin lesions in psoriatic mice altered by S100a4^−/−^

To elucidate the effect of S100A4 on the pathogenesis of psoriasis, we first generated two gRNAs to target the mouse S100a4 gene by CRISPR/Cas9 gene editing (**Supplementary Figure S1A**), producing the founder lines with the mutation. Genotyping PCR confirmed the absence of full-length transcripts in homozygous mice for the KO allele (hereafter referred to as S100a4**^−^**^/^**^−^**) after passaging, indicating successful targeting of the S100a4 gene (**Supplementary Figures S1B, C**). Western blot analysis further demonstrated the absence of S100a4 protein in skin tissues from adult S100a4**^−^**^/^**^−^** mice (**Supplementary Figure S1D**). To characterize psoriasis pathology, we applied IMQ continuously to the dorsal skin of mice to induce and construct psoriasis models, while the Sham-operated group was coated with Vaseline as a control (**Supplementary Figure S1E**). Mice of all genotypes in this study developed normally, and there was no significant change in body weight (**Supplementary Figure S1F**), but a higher degree of skin erythema, scaliness, and skin thickness was observed in both psoriatic mice compared to the controls. When compared with the WT psoriasis mice, these indicators were significantly lower in the KO group (**Supplementary Figure S1G**). Strikingly, by comparing the psoriasis area and severity index (PASI), we found that the PASI of S100a4**^−^**^/^**^−^** mice was significantly lower than that of WT mice (**Supplementary Figure S1H**), suggesting that KO S100a4 largely attenuated psoriasis. H&E staining also showed that KO S100a4 alleviated epidermal thickening and inflammatory cell infiltration due to psoriasis to a certain extent (**Supplementary Figure S1I**).

**Figure 1:**
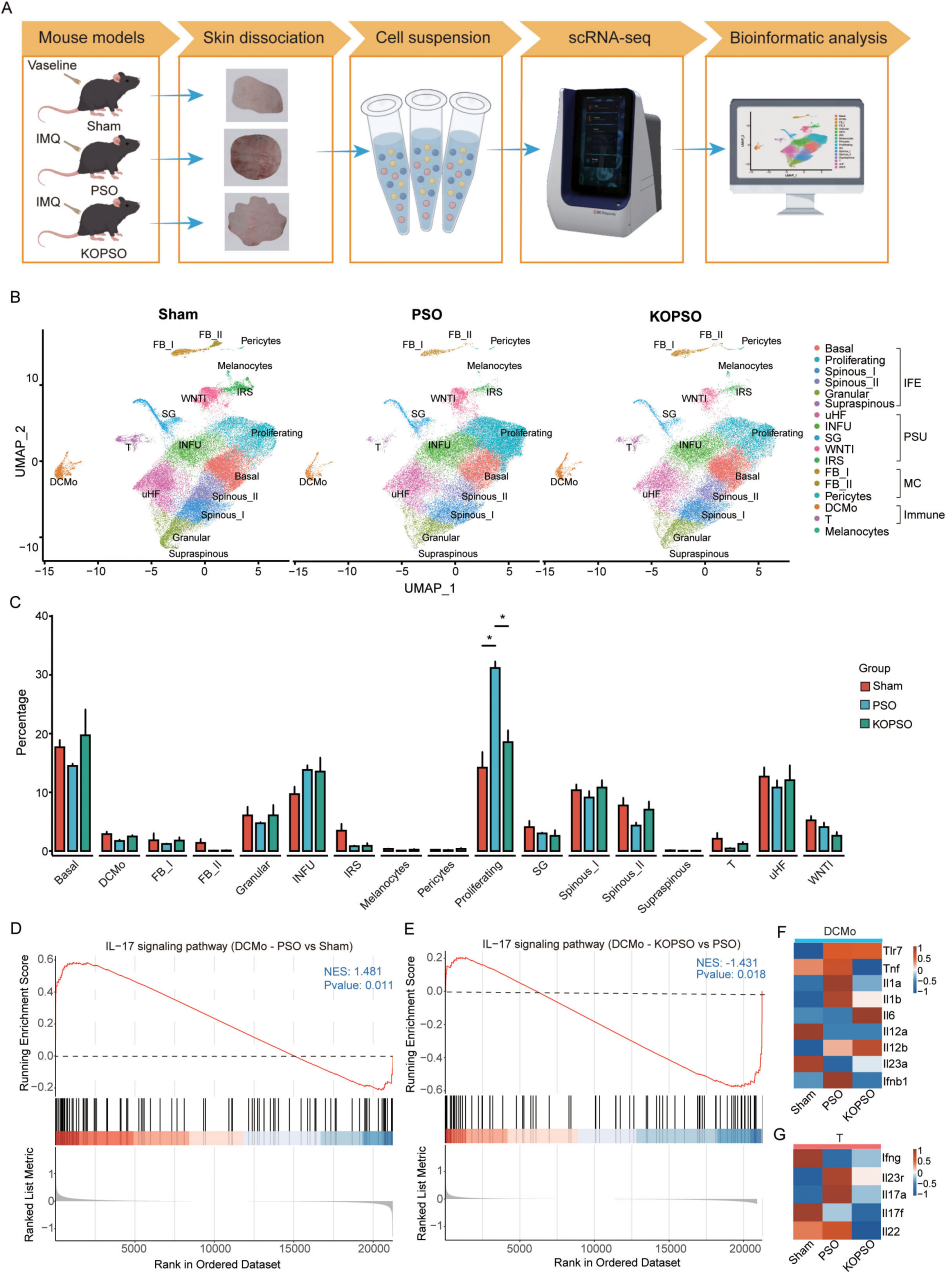
ScRNA-seq analysis of all cells in Sham-operated, psoriasis, and S100a4^−/−^ psoriasis mice. **(A)** Schematic diagram of the experimental design. **(B)** UMAP plot of single-cell transcriptomic profiles from Sham, PSO, and KOPSO samples. Colors indicate annotations. **(C)** Bar plot comparing the proportions of cell populations of each cell type within each sample group. Statistical analysis was performed using the Student’s *t*-test. *p < 0.05. **(D)** GSEA of the “IL-17 signaling pathway” in DCMo from PSO *vs*. Sham. **(E)** GSEA of “IL-17 signaling pathway” in DCMo from KOPSO *vs*. PSO. NES, normalized enrichment score. **(F)** Heatmap showing the expression profile of selected genes in DCMo. **(G)** Heatmap showing the expression profile of selected genes in T cells.

To continue exploring the effect of KO S100a4 on the transcriptional landscape of skin-resident cells, we further collected dorsal skin tissues from control (Sham), model (PSO), and S100a4**^−^**^/^**^−^** group (KOPSO) mice for scRNA-seq (BD Rhapsody platform) (**Figure 1A**). After the initial quality control and removal of doublets (see Materials and Methods for details), we obtained a total of 138,115 single-cell transcriptome profiling data and annotated the cell types based on many known cell-specific markers, covering major cells of the interfollicular epidermis (IFE) and pilosebaceous unit (PSU), as well as mesenchymal cells (MCs), melanocytes, and immune cells, which were further distinguished into 17 cell clusters (**Figure 1B**; **Supplementary Figure S2A**). In IFE cells, six KC subpopulations were identified: one basal cluster, one proliferating cluster, two spinous layer clusters (spinous I and II), and granular layer and supraspinous clusters with similar markers. In PSU cells, five cell clusters were identified, including sebaceous gland (SG) cells linked to SGs, upper hair follicle (uHF) cells and infundibulum (INFU) cells located in the upper layer of the hair follicle, and inner root sheath (IRS) cells representing the innermost layer of the hair follicle, as well as WNTI clusters involved in wingless (WNT) signaling inhibition. WNTI and these follicular subpopulations represent the major interfacial zones between the skin epithelium and the environment and may play an important role in hair follicle development and barrier function. Likewise, MCs were divided into three clusters including two fibroblast (FB) subpopulations (FB I and II) and pericytes, where FB I and FB II fibroblasts specifically express Pi16 and Col15a1, respectively, and are considered to be the main source of all specialized or disease-specific fibroblasts. Additionally, two types of immune cell clusters were identified, one cluster of Cd3-expressing T lymphocytes and another specific cell type with high expression of Cd74 and MHC class II molecules, possibly dendritic cell (DC)-derived monocyte population (DCMo), and in terms of functional attributes, the T cells mainly mediate adaptive immune response, whereas the DCMo has more preference to participate in antigen processing and presentation, favoring DC properties, thereby initiating and regulating the above behavior, but in any case, they all play an integral role in the immune defense system in psoriasis.

It is well known that KC abnormalities like hyperproliferation, hyperkeratosis, and parakeratosis are the most prominent clinical phenotypes in psoriasis; however, by comparing the percentage of each cell type in the resident skin, we found that proliferating KCs were significantly increased in the PSO group, in contrast to almost no change or even a downward trend for the cells in the basal and differentiated layers (e.g., spinous and granular), and it is noteworthy that proliferating KCs by nature also originated from basal KC clusters but abnormally (high expression of Krt14 and Krt5 as well as high levels of cyclins, see **Supplementary Figure S2A**); these findings suggest that PSO preferentially induces excessive proliferation of basal KCs in mice, which is likely the main culprit causing epidermal thickening, whereas the KOPSO group reversed this process (**Figure 1C**), implying that KO S100a4 largely rescued the aberrant proliferative phenotype of KCs. Notably, S100a4 might behave directly on immune cells, and they had an obvious immunity restorative effect after KO (**Figure 1C**; **Supplementary Figure S2B**), possibly contributing to the alleviation of immune inflammation. To shed light on this process, we further executed GSEA, and results revealed that in DCMo cells, PSO activated the Toll-like receptor signaling pathway and the IL-17 signaling pathway in addition to eliciting strong antigen processing and presentation (**Figure 1D**; **Supplementary Figure S2C**), and interestingly, KOPSO significantly inhibited the IL-17 signaling pathway (**Figure 1E**), suggesting that S100a4**^−^**^/^**^−^** may be effective in ameliorating the chronic inflammatory response in psoriasis by repressing this signaling axis. IMQ, a TLR7 agonist, applied independently rapidly induces a pathology closely resembling human psoriasis and is known to stimulate DC activation via TLR7 signaling, thereby mediating pathogenic initiation, as confirmed in our study, although we also found that S100A4**^−^**^/^**^−^** did not appear to alter IMQ-mediated Tlr7 activation but reversed the expression of many pro-inflammatory cytokines as well as members of the type I interferon (IFN-I) family such as Ifnb1 in DCMo (**Figure 1F**), which is usually implicated in the differentiation and function of T helper (Th) cells such as Th1 (producing Ifng) and Th17 (producing IL-17), and furthermore, the gene expression profiles from T cells showed that S100a4**^−^**^/^**^−^** strongly remedied the PSO response to the IL-23/IL-17 axis instead of Ifng (**Figure 1G**), whereby S100A4**^−^**^/^**^−^** may largely remodel the classical IL-23/IL-17 signaling axis induced by IMQ-mediated psoriasiform dermatitis.

### S100a4 knockout remodels the multicellular landscape and powerfully reprograms proliferating keratinocytes in psoriasis

**Figure 2:**
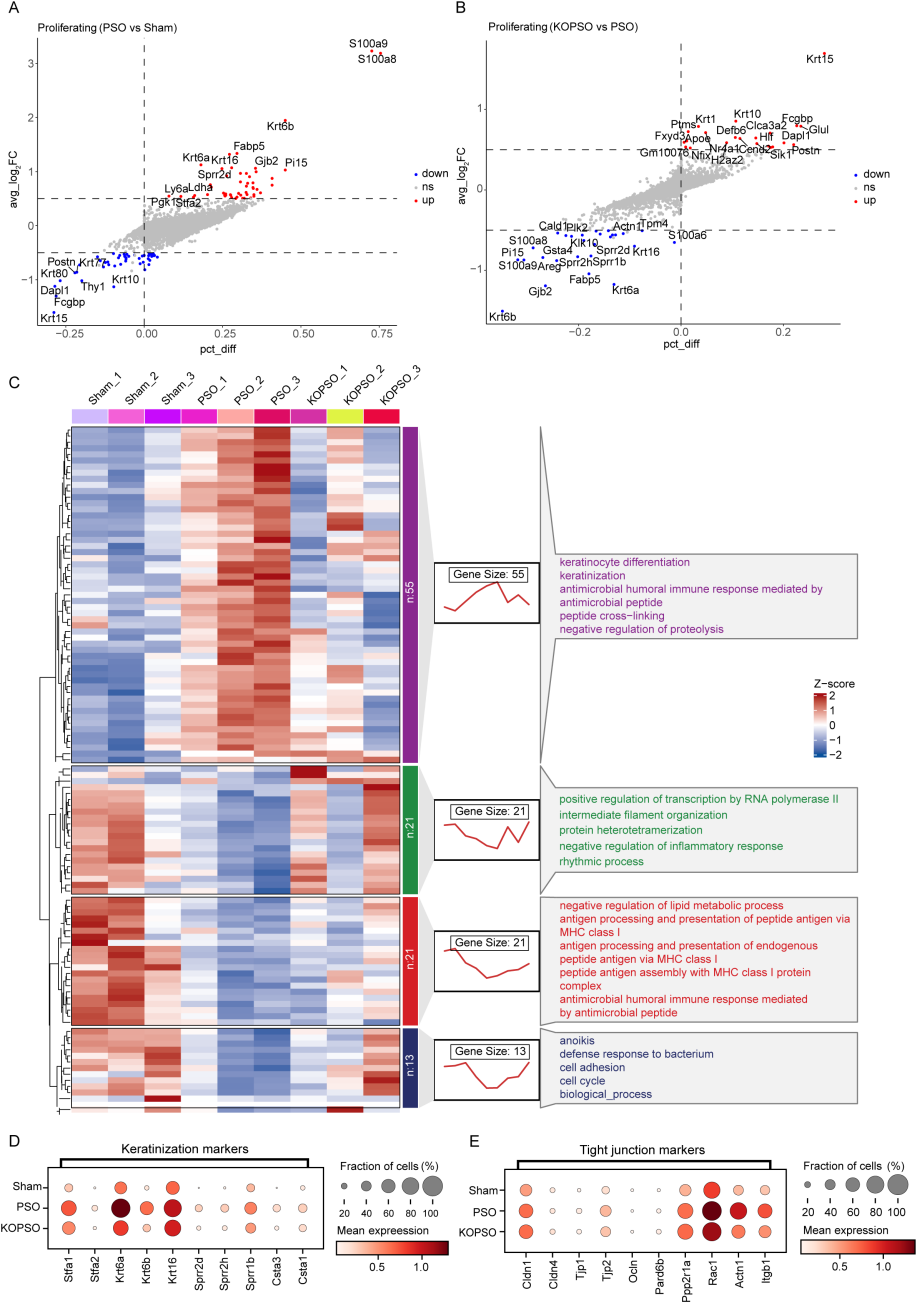
Identification of gene expression profiles in proliferating KCs associated with psoriasis. **(A)** Volcano plot showing all DEGs between PSO and Sham samples in proliferating KCs. **(B)** Volcano plot showing all DEGs between KOPSO and PSO samples in proliferating KCs. **(C)** Hierarchical clustering heatmap and line chart displayed the clustering patterns and trends of DEGs in proliferating KCs in three pairs of comparison groups, and exhibited the top 5 enriched GO pathways in each cluster. **(D)** Dot plot showing expression of keratinization markers in each group. **(E)** Dot plot showing expression of tight junction markers in each group.

Having established the critical role of S100a4 in psoriasis, we sought to map its functional impact at single-cell resolution. We first systematically evaluated the transcriptomic alterations induced by S100a4 KO across all identified cell clusters, which revealed a broad recalibration of both immune and stromal compartments. Specifically, PSO drastically induced strong transcriptomic changes across all cell populations in the microenvironment, notably promoting the significant upregulation of genes associated with keratinization and the regulation of cell proliferation (**Supplementary Figures S3A, B**). In contrast to PSO, S100a4**^−^**^/^**^−^** largely remodeled the transcriptional landscape of multiple cell clusters, with a particularly profound alteration in the transcriptional profiles of KC subpopulations. This included potent suppression of keratinization-related gene expression and, more importantly, marked inhibition of genes involved in immune response and chemotaxis (**Supplementary Figures S3C, D**), suggesting a potential reduction in signal intensity for immune cell recruitment and thereby attenuated inflammatory disruption. As the predominant cell type in the skin epidermis, KCs have been shown to be closely associated with psoriasis pathogenesis (8). Having described previously that PSO induced dramatic proliferation of proliferating KCs, here we went on to investigate the gene expression signatures in proliferating KCs (**Figure 2A**); specifically, a total of 110 DEGs were determined among the three groups, in which PSO resulted in significant upregulation of a large number of genes related to KC differentiation or keratinization (**Supplementary Figure S3B**), whereas S100a4**^−^**^/^**^−^** significantly suppressed the expression of these genes (**Figure 2B**; **Supplementary Figure S3D**), which might have prevented the hyperkeratosis of the skin to some extent.

Based on the heterogeneity of the expression of all DEGs in the samples, they could also be classified into four different gene expression patterns with superior inter-sample dependency trends and might be involved in different biological functions; as expected, the significantly upregulated genes in PSO remained in the same expression pattern associated with skin keratinization, but their expression was suppressed to varying degrees after KO S100a4. Relatively, DEGs significantly downregulated in PSO exhibited three different expression patterns, mainly associated with transcriptional regulation, intermediate filaments, rhythms, MHC-like antigen processing, and cell adhesion, indicating that PSO might also be regulated by these processes as a result of multifactorial action, and that perhaps S100a4**^−^**^/^**^−^** could also improve psoriasis symptoms by moderating the expression of these genes, especially those linked to transcriptional regulation, intermediate filaments, and rhythmic processes (**Figure 2C**). Moreover, we concretely examined the expression of genes related to keratinization and skin barrier in proliferating KCs, focusing on significant alterations in the type II keratins Krt6a and Krt6b, as well as considerable changes in the expression of scaffolding proteins linking the tight junction (TJ), such as Cldns and Tjps, and cytoskeletal protein Actn1, and found that PSO markedly promotes the expression of keratinized and TJ-associated markers, which may lead to epidermal thickening and disruption of skin barrier integrity, yet in the KOPSO group, these alterations were moderately controlled (**Figures 2D, E**), reflecting the remarkable regulatory capacity of S100a4**^−^**^/^**^−^** in restoring skin homeostasis.

### Effects of S100a4^−/−^ on proliferation and differentiation dynamics of keratinocytes in psoriatic mice

**Figure 3:**
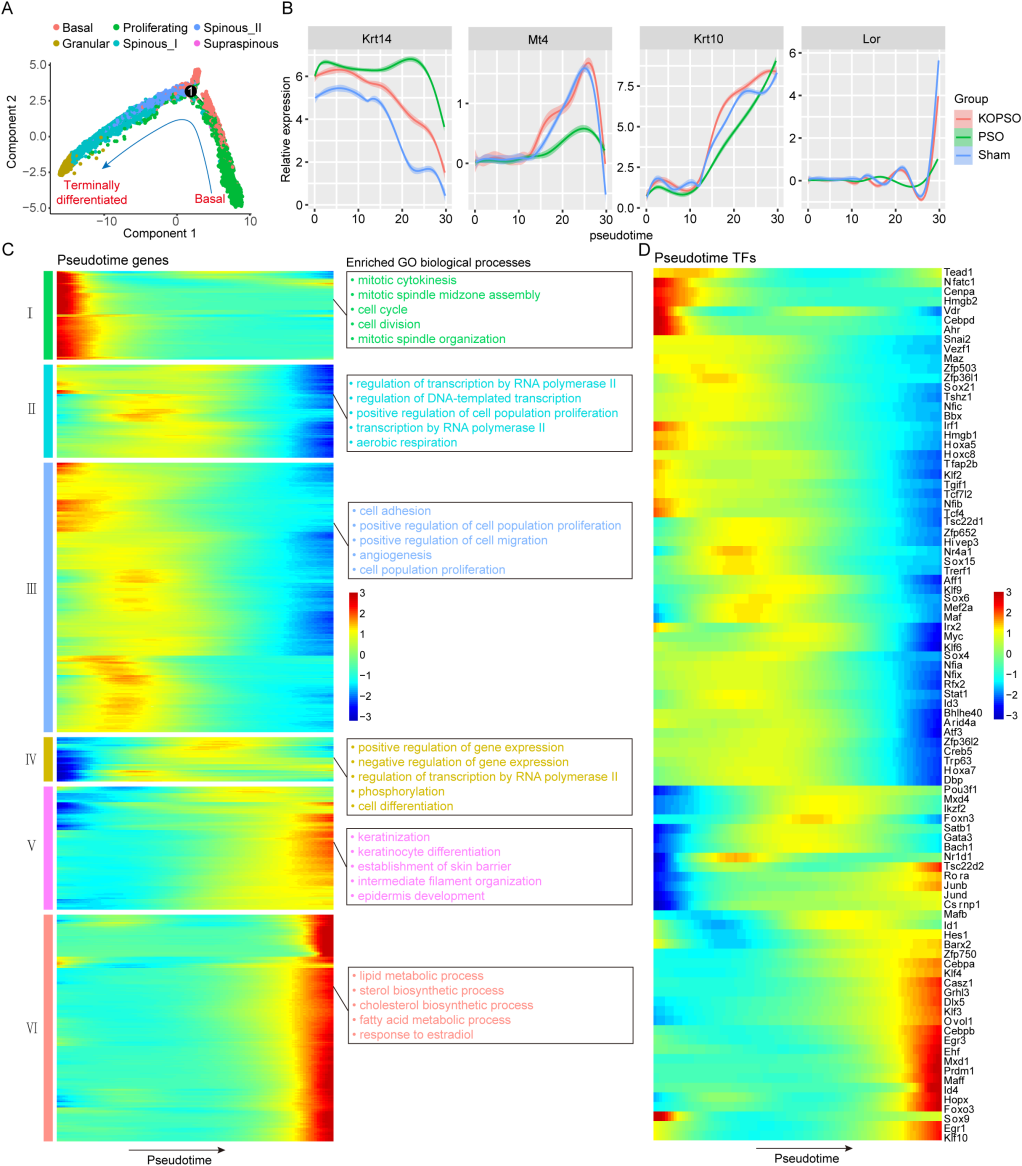
Dynamic process of KCs proliferation and differentiation in psoriasis. **(A)** KCs are colored by cell type and ordered along pseudotime progression. **(B)** The line plots showing the pseudotemporal expression trend of stage markers in each group. **(C)** Heatmap showing the spline-smoothed expression pattern of pseudotime-dependent genes along with the GO enrichment terms for distinct gene clusters. **(D)** Heatmap showing the pseudotemporal expression of transcription factor.

KC cells play a key role in securing the skin barrier function, and their constant renewal reduces the risk of infection and scar formation and maintains the skin in a healthy state. To dissect the potential effects of S100a4**^−^**^/^**^−^** on the proliferation and differentiation dynamics of psoriatic KC cells, we next reconstructed a differentiation trajectory along pseudotime by ordering all KC cells, which contains the whole range of basal to terminally differentiated KC cells. Although pseudotime analysis raised the expectation from the basal to the granular layer, KC dynamics still showed many conspicuous differences prior to terminal differentiation, particularly a sudden shift in proliferating KCs at earlier periods of pseudotemporal time that may be related to a specific state (**Figure 3A**; **Supplementary Figure S4A**). The expression of some well-recognized markers Krt14 (basic), Mt4 (transient), Krt10 (mature), and Lor (terminally differentiated) at different differentiation phases of KC cells along the defined pseudotemporal axis confirmed that our cell alignment was correct, and importantly, we found that the pseudotemporal-dependent expression of Mt4 showed extremely visible intergroup differences between the PSO group and the other two groups (**Figure 3B**). Mt4 is a transient marker possibly marking a transitory stage (38), that is to say, PSO likely led to the abnormal differentiation of transitional KC cells.

Next, we identified 1,126 pseudotime-dependent genes with significant differences and clustered them into six groups (I–VI) according to their expression patterns during the differentiation process (**Figure 3C**), representing different periods of differentiation also associated with different functional characteristics. For example, in the early stage (I), these genes were mainly involved in mitosis, cell cycle, and cell division, defined as basal features, and then in the transition stage (II–IV), transcriptional activities become active and genes linked to proliferation and development gradually come to the forefront, followed by the maturation stage (V), where genes related to intermediate filament organization, KC differentiation, keratinization, establishment of skin barrier, and epidermis development were clearly observed to rapidly predominate, and finally, at the end of terminal differentiation (VI), the anterior wave of genes begins to slowly diminish, and was replaced by the expression of genes related to lipid and fatty acid metabolism, sterol, and cholesterol biosynthesis, which provide essential functional support to the skin (**Supplementary Figure S4B**). Afterwards, to gain insight into the molecular regulation of PSO- or S100a4**^−^**^/^**^−^**-mediated epidermal differentiation, we also explored the expression patterns of all pseudotime-dependent transcription factors (TFs) during differentiation, and found that only a few TFs functioned in either early or late stages, while a larger number of TFs were found in the transition stage (**Figure 3D**). This finding suggests that S100a4**^−^**^/^**^−^** may play a positive role in correcting the PSO-induced abnormalities of the transition from basal to proliferating KC cells.

### S100a4^−/−^ orchestrates RNA regulatory network misled by psoriasis in proliferating keratinocytes

**Figure 4:**
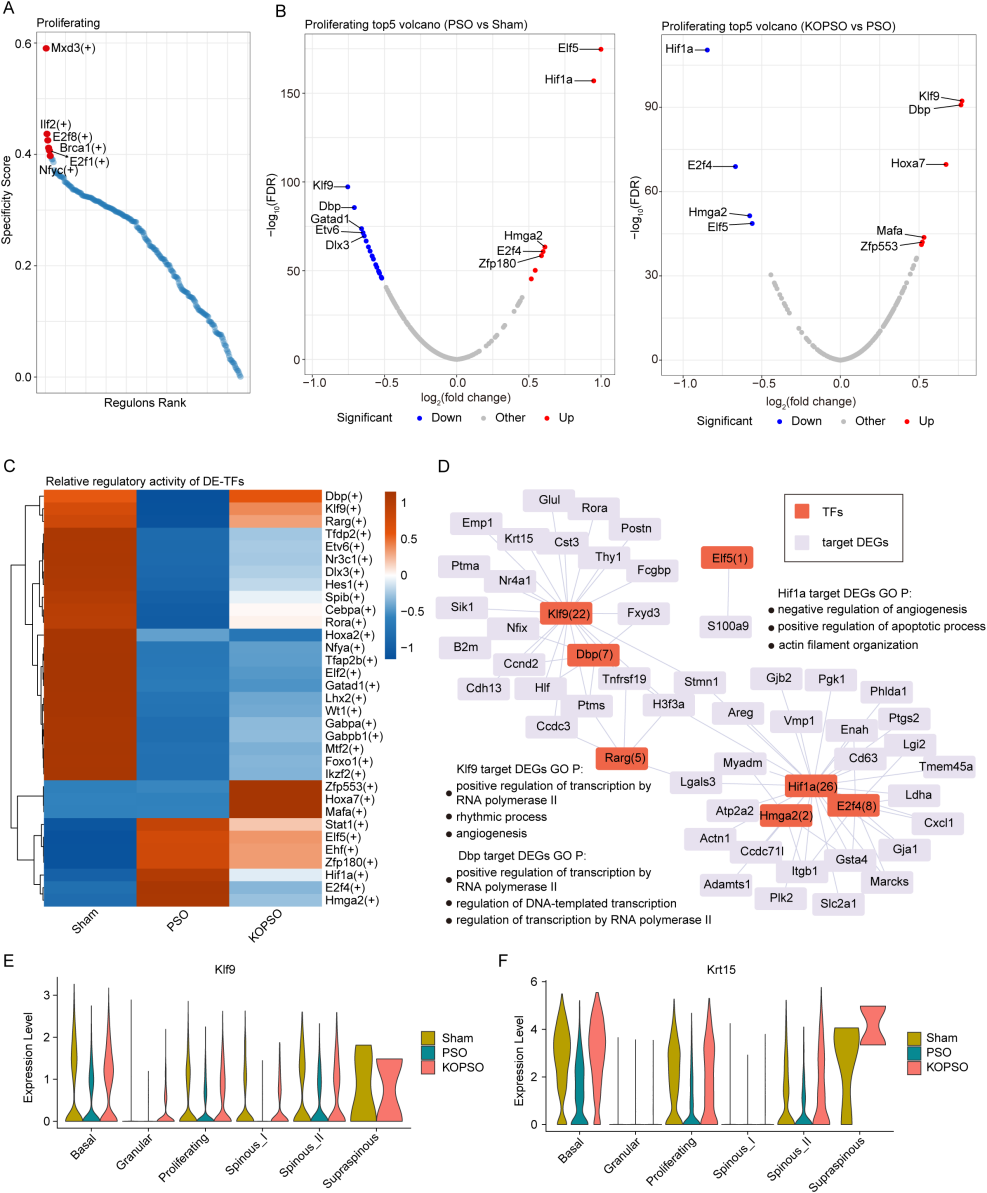
Dysregulation of regulons related to KCs. **(A)** Regulon rank of proliferating KCs. The six with the most specificity are marked. **(B)** The volcano plot showing differential transcription regulons in proliferating KCs. Blue represents downregulation, red represents upregulation. **(C)** The heatmap showing the relative regulatory activity changes of the identified DE-TFs across three groups. **(D)** Network showing that seven DE-TFs and their target DEGs in proliferating KCs. **(E, F)** Violin plot showing the expression levels of Klf9 and Krt15.

To clarify the key transcriptional regulatory mechanisms linked to psoriatic KC dynamics, we sought to identify and characterize vital regulons specifically activated in proliferating KCs that fluctuated significantly or had possible transition disorders in PSO, among them some previously described ones related to epidermal development such as Ilf2(+) (39), but also some new ones, such as Mxd3(+) (**Figure 4A**). In addition, a total of 33 significantly differentially expressed TFs (DETFs) were identified, and it is worth mentioning that the KOPSO group significantly reversed the misled expression of 6 DETFs induced by PSO, namely, Elf5, Hif1a, E2f4, Hmga2, Klf9, Dbp, and Rarg (**Figure 4B**). Correspondingly, the relative regulatory activities of these DETFs were also recovered to near-normal control levels (**Figure 4C**). Elf5, as the most significant DETF in PSO, has been confirmed to play an important role in regulating KC proliferation and differentiation (40), and Hif1a, as the most prominent DETF in KOPSO, is important for controlling angiogenesis and skin inflammation, as well as being a key effector in inhibiting terminal differentiation and increasing KC proliferation (41). Remarkably, Klf9 and Dbp were S100a4**^−^**^/^**^−^** strongly reversed DETFs that were significantly downregulated in PSO, and both were involved in rhythm regulation (42, 43); in addition, they also showed pseudotime-dependent expression during the KC transition (see **Figure 3D**), which might mediate the regulation of the proliferation–differentiation axis at the KC transition stage.

To further prove this point, we constructed cellular transcriptional regulatory networks of DETFs and their target DEGs (**Figure 4D**). Based on the target DEGs regulated by TFs and the corresponding regulatory importance scores, we found that Hif1a may act as an important regulator of gap junction-associated Gja1 expression to mediate hyperadhesion between KCs (described in the next section). More importantly, Klf9 potently regulates the expression of Krt15 (score ranked 1st out of 22 candidates), an intermediate filament protein responsible for the structural integrity of KC cells, which was also found to be one of the several most significantly DEGs in proliferating KCs (**Figure 2B**), indicating that the potential transcriptional regulatory impact of Klf9 on Krt15 may contribute substantially to the maintenance of normal cellular structure and function. Moreover, not only in basal and proliferating KCs, but also in differentiated KCs, was Klf9 and Krt15 expression in PSO significantly misregulated (**Figures 4E, F**), highlighting that the Klf9–Krt15 axis imbalance may largely determine cell fate as well. These further demonstrate that Klf9 may influence the proliferation/differentiation of KCs at the transition period by controlling Krt15 expression in a rhythm-dependent manner, yet this process appears to be brightly orchestrated in the case of KO S100a4.

### S100a4^−/−^ rescues the cellular abnormalities of keratinocytes by monitoring cell–cell crosstalk

**Figure 5:**
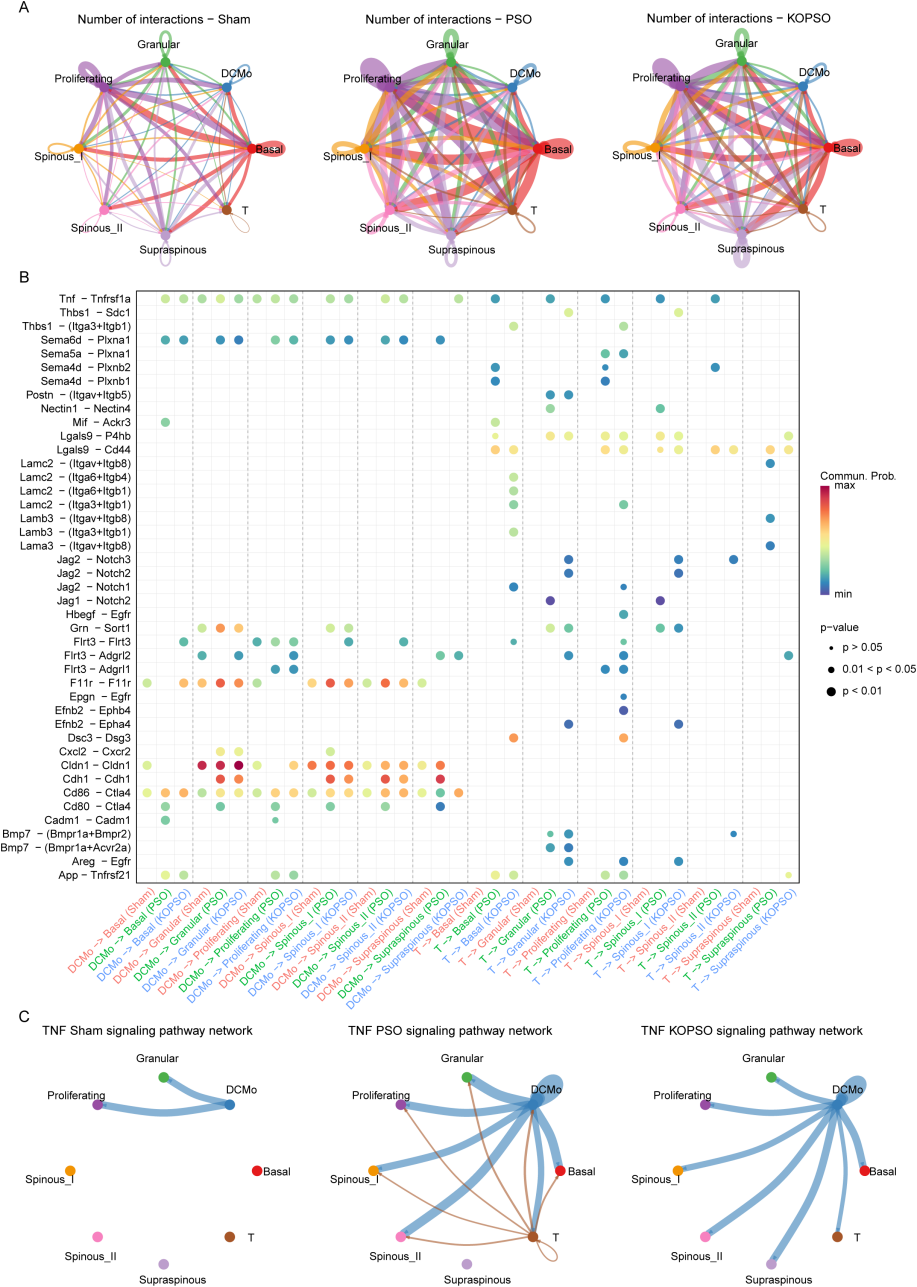
S100a4^−/−^ rescues keratinocytes by monitoring cell–cell interactions. **(A)** The circular plot showing the number of interactions in the Sham, PSO, and KOPSO samples between cell types. **(B)** The bubble plot showing the signaling pathways from DCMo and T cells to six types of keratinocytes in the Sham, PSO, and KOPSO samples. **(C)** The circular plot showing the TNF signaling pathway between cell types in the Sham, PSO, and KOPSO samples.

Within the context of psoriasis, communication between immune cells and KC cells not only is critical for maintaining skin homeostasis, but also plays an integral role in skin inflammation and healing processes. To deepen the understanding of the complex interactions among various cell populations, here we performed an extensive analysis of the intercellular interactions between each KC cell type and with immune cells. It was found that PSO drove a dramatic expansion of the interactions information between all cell populations in contrast to the Sham group, whether between distinct KC cells or with DCMo or with T cells, which also implies that PSO forcefully stimulated the immune response overload in skin tissue. Excitingly, it was partially converged in the KOPSO group, particularly in terms of basal and proliferating KCs, emphasizing their critical positions in the cellular response hierarchy (**Figure 5A**). To further clarify the external incoming signaling flow that may contribute to KC abnormalities, based on inferred ligand–receptor pair changes, we purposed a presumably involved key inflammatory pathway (Tnf-Tnfrsf1a). This pathway is markedly augmented in PSO but attenuated or vanished in KOPSO, especially for interplay with the T cells (**Figures 5B, C**). Thus, it is suspected that S100A4**^−^**^/^**^−^** facilitates KC survival and inflammation resolution by blocking this signaling pathway and then reducing recruitment and killing from T cells. This aside, we also found that the T cell-mediated Notch signaling pathway might be important for differentiated KCs, mainly keyed by ligands Jag1 and Jag2 secreted by T cells, and in addition, cell adhesion-related signals between DCMo and KCs (e.g., F11r, Cldn1, and Cdh1) were found to be enhanced in both PSO and KOPSO, which appeared to be linked to exogenous IMQ stimulation, and this enhanced adhesion signaling led to rapid recruitment of DCMo in the lesion area, thereby causing local immune activation (**Figure 5B**).

For general and exceptional interactions received by proliferating cells within KCs, we found that signaling pathways related to epidermal homeostasis such as WNT, Notch, bone morphogenetic protein (BMP), and receptor tyrosine kinase (RTK) were not functioning properly in the PSO; in addition, those pathways related to cell adhesion, extracellular matrix (ECM) components, and semaphorins were abetted as well; just encouragingly, we noticed that among the pathways involved in cell adhesion, the gap junction component Gja1 and desmosome member desmocollins, which mediated pathways, were greatly influenced by S100a4**^−^**^/^**^−^** intentionality (**Supplementary Figure S5**), and that the massive enhancement of both gap junctions and desmosome intercellular junctions could propel KCs to respond rapidly to external stimuli and provide stronger mechanical support, contributing to cellular hyperadhesion and shear thickening, while S100a4**^−^**^/^**^−^** reduced immune stimulation possibly due to slashed release of inflammatory mediators, thus contributing to the re-establishment of normal KC intercellular junctions and balancing cell–cell crosstalk.

## Discussion

The cellular composition of the psoriatic epidermis has been extensively studied in recent decades. Investigations using mouse models have garnered increasing interest, enriching their utility for basic research, preclinical modeling, and translational medicine in psoriasis (44). In this study, our data suggest an important role for S100A4 using IMQ murine models and highlight its potential cell-specific transcriptomic control of immunoinflammation and epidermal hyperplasia in psoriasis, implying its active involvement in disease pathogenesis. Our results showed that KO S100a4 effectively moderated epidermal thickening and inflammatory infiltration induced by IMQ, corroborating previous adverse observations of significantly high S100A4 expression in psoriatic skin (13). To explore the molecular function of S100a4 *in vivo*, we employed scRNA-seq to comprehensively dissect cellular heterogeneity and examined various cellular states in psoriatic mouse skin. Our analyses revealed that S100a4**^−^**^/^**^−^** markedly influenced immunomodulation-related signaling pathways and KC proliferation/differentiation dynamics. We also observed dysregulation of cellular transcriptional regulatory network, likely related to continuous excessive proliferation. Together, these findings provide subtle new insights into how psoriatic skin-resident cell development experiences a highly dynamic process of transcriptome transformation.

DCs are understood to play a key role in the etiology and immune dysregulation of psoriasis, with studies revealing distinct anomalies in DC subpopulations during the initial stages of psoriasis. Briefly, psoriasis-activated plasmacytoid DCs (pDCs) are known to promote myeloid DCs (mDCs) maturation via IFN-I signaling, and the latter subsequently produce TNF-α, IL-12, and IL-23 to drive the differentiation of Th1 and Th17 cell subsets, with IL-23 being pivotal in Th17 production. Afterwards, Th17 cells secrete multiple inflammatory cytokines, notably IL-17, which activates KCs to produce AMPs, cytokines, and chemokines. These molecules in turn recruit more immune cells to enlarge the inflammatory response (4, 45, 46). Consistent with this paradigm, our scRNA-seq data indicated an increase in the IFN-I signaling member Ifnb1, the cytokine Tnf, and the IL-17 signaling pathway marker Il17a during psoriasis progression, but attentively, KO S100a4 suppressed the expression of this entire axis. Admittedly, our analysis results further highlighted that cytokines secreted from immune cells also appear to mediate crosstalk with KCs, like free Tnf could interact with the KC surface receptor Tnfrsf1a, thereby activating an intracellular cascade of responses, which is usually inextricably linked to cell survival, apoptosis, and inflammation (47, 48). The observed reduction in Tnf expression upon S100a4 deletion could therefore attenuate this intercellular communication, potentially limiting excessive inflammatory infiltration and KC injury. Taken together, our data are consistent with enhanced IFN-I signaling and inflammatory immune cell activation following IMQ induction, processes implicated in psoriasis occurrence. S100a4 KO may ameliorate pathology, at least in part, by inhibiting the expression of key inflammatory mediators, thereby potentially affecting immune cell migration and localization. This discovery underscores the critical role of S100A4 in regulating immune responses and suggests that it may be an important target for future therapeutic interventions.

The main clinical manifestations of psoriasis are evident at the outermost layer of the skin, which is made up of KCs. In the process of interpreting the heterogeneity of KCs, we discovered that a subtype of proliferating KCs increased significantly in the psoriasis group, but decreased obviously after KO S100a4. This finding indicated that S100a4**^−^**^/^**^−^** might play an essential role in suppressing the abnormal proliferation of psoriatic KCs. A previous study found that silencing S100a4 in xenograft SCID mouse models significantly reduced epidermal thickness and dermal vascularization, and impaired KCs proliferation, which is consistent with our results (13). More interestingly, in our scRNA-seq analysis, S100a4**^−^**^/^**^−^** might help re-establish the differentiation trajectory of basal KCs. Although their likelihood to undergo appropriate proliferation and differentiation declines with pathological exacerbation in psoriasis, these cells retain a degree of plasticity and can provide long-term renewal capacity. During the transitory stage of KCs, we noted the re-stabilization of transcriptional regulatory network in proliferating KCs from S100a4**^−^**^/^**^−^** psoriatic mice. Some TFs of interest, like Elf5, Hif1a, E2f4, Hmga2, Klf9, Dbp, and Rarg, are worthy of attention, along with their potentially relevant pathways such as rhythms and angiogenesis. In addition, several lines of evidence indicate that they may be involved in the process of aberrant proliferation and keratinization of KCs (40–43, 49–51). In addition to identifying conserved networks and pathways, our analysis also identified a number of target genes that may play key roles and hold promise as candidates for future psoriasis studies; for example, Krt15, as a marker expressed by epidermal basal KCs and hair follicle stem cells, is essential for the maintenance of skin homeostasis, and it is also required for KC differentiation (52–54). Gja1 is a highly abundant member of the connexin family expressed in the epidermis and plays a vital role during continuous renewal of the epidermis, and its mediated signaling alteration via connexin channels has been treated as a key contributor to the psoriatic state (55–57). The above results stressed the importance of S100a4**^−^**^/^**^−^** in KC dynamic processes and psoriasis pathogenesis, at least partly, by suppressing KC hyperproliferation and abnormal differentiation at inflamed skin lesions.

Notwithstanding these insights, our study has certain limitations. The interpretations concerning cell–cell communication and KC fate dynamics primarily rely on inferred computational analyses, such as CellChat-based ligand–receptor interactions and pseudotime trajectory reconstruction. Although these methods provide valuable insights, their conclusions must be interpreted with caution. CellChat predictions depend on curated ligand–receptor databases and expression thresholds to suggest potential signaling events, but do not confirm physical interactions or spatial proximity. Similarly, pseudotime trajectories are reconstructions based on transcriptional similarity and are sensitive to dimensionality reduction and clustering parameters; even when establishing a plausible model of developmental continuity, they do not represent a definitive temporal order. Consequently, the cellular interactions and differentiation pathways proposed herein should be regarded as testable hypotheses rather than established mechanisms. Future experimental validations will be essential to confirm these predictions and to elucidate the precise mechanisms through which S100a4 deficiency modulates KC behavior in psoriasis.

## Conclusions

Collectively, we have generated a large resource of single-cell gene expression profiles from mouse skin and used it to dissect epidermal heterogeneity. Our data provided compelling evidence that KO of S100a4 effectively mitigated the skin lesion phenotype and inflammatory response in mouse models exhibiting psoriasis-like symptoms induced by IMQ. Furthermore, our investigation identified a noteworthy association between S100A4 and the underlying pathomechanism of psoriasis. Despite the limitations of mouse models in predicting human disease, our results provide a potential explanation for clinical observation that high S100A4 expression in psoriasis patients is highly correlated with psoriatic flares. Our findings provide insights into the functional roles of S100A4 in psoriasis and offer novel avenues for therapeutic exploration and intervention guidance in this disease.

## Data Availability

The original contributions presented in the study are publicly available. This data can be found here: https://www.ncbi.nlm.nih.gov/geo/ under accession number GSE312644.

## References

[1] Griffiths CEM Armstrong AW Gudjonsson JE Barker J . Psoriasis. Lancet. (2021) 397:1301–15. doi: 10.1016/S0140-6736(20)32549-6, PMID: 33812489

[2] Raharja A Mahil SK Barker JN . Psoriasis: a brief overview. Clin Med (Lond). (2021) 21:170–3. doi: 10.7861/clinmed.2021-0257, PMID: 34001566 PMC8140694

[3] Armstrong AW Read C . Pathophysiology, clinical presentation, and treatment of psoriasis: A review. JAMA. (2020) 323:1945–60. doi: 10.1001/jama.2020.4006, PMID: 32427307

[4] Rendon A Schakel K . Psoriasis pathogenesis and treatment. Int J Mol Sci. (2019) 20:1475. doi: 10.3390/ijms20061475, PMID: 30909615 PMC6471628

[5] Tokuyama M Mabuchi T . New treatment addressing the pathogenesis of psoriasis. Int J Mol Sci. (2020) 21:7488. doi: 10.3390/ijms21207488, PMID: 33050592 PMC7589905

[6] Lee HJ Kim M . Challenges and future trends in the treatment of psoriasis. Int J Mol Sci. (2023) 24:13313. doi: 10.3390/ijms241713313, PMID: 37686119 PMC10487560

[7] Yu J Zhao Q Wang X Zhou H Hu J Gu L . Pathogenesis, multi-omics research, and clinical treatment of psoriasis. J Autoimmun. (2022) 133:102916. doi: 10.1016/j.jaut.2022.102916, PMID: 36209691

[8] Zhou X Chen Y Cui L Shi Y Guo C . Advances in the pathogenesis of psoriasis: from keratinocyte perspective. Cell Death Dis. (2022) 13:81. doi: 10.1038/s41419-022-04523-3, PMID: 35075118 PMC8786887

[9] Orzan OA Tutunaru CV Ianosi SL . Understanding the intricate pathophysiology of psoriasis and related skin disorders. Int J Mol Sci. (2025) 26:749. doi: 10.3390/ijms26020749, PMID: 39859462 PMC11766135

[10] Capon F . The genetic basis of psoriasis. Int J Mol Sci. (2017) 18:2526. doi: 10.3390/ijms18122526, PMID: 29186830 PMC5751129

[11] Dascalu RC Barbulescu AL Stoica LE Dinescu SC Bita CE Popoviciu HV . Review: A contemporary, multifaced insight into psoriasis pathogenesis. J Pers Med. (2024) 14:535. doi: 10.3390/jpm14050535, PMID: 38793117 PMC11122105

[12] Liang H Li J Zhang K . Pathogenic role of S100 proteins in psoriasis. Front Immunol. (2023) 14:1191645. doi: 10.3389/fimmu.2023.1191645, PMID: 37346040 PMC10279876

[13] Zibert JR Skov L Thyssen JP Jacobsen GK Grigorian M . Significance of the S100A4 protein in psoriasis. J Invest Dermatol. (2010) 130:150–60. doi: 10.1038/jid.2009.206, PMID: 19641515

[14] Mitoma C Kohda F Mizote Y Miake A Ijichi A Kawahara S . Localization of S100A2, S100A4, S100A6, S100A7, and S100P in the human hair follicle. Fukuoka Igaku Zasshi. (2014) 105:148–56. 25507257

[15] Ambartsumian N Klingelhofer J Grigorian M . The multifaceted S100A4 protein in cancer and inflammation. Methods Mol Biol. (2019) 1929:339–65. doi: 10.1007/978-1-4939-9030-6_22, PMID: 30710284

[16] Garrett SC Varney KM Weber DJ Bresnick AR . S100A4, a mediator of metastasis. J Biol Chem. (2006) 281:677–80. doi: 10.1074/jbc.R500017200, PMID: 16243835

[17] Schneider M Hansen JL Sheikh SP . S100A4: a common mediator of epithelial-mesenchymal transition, fibrosis and regeneration in diseases? J Mol Med (Berl). (2008) 86:507–22. doi: 10.1007/s00109-007-0301-3, PMID: 18322670

[18] O'Reilly S . S100A4 a classical DAMP as a therapeutic target in fibrosis. Matrix Biol. (2024) 127:1–7. doi: 10.1016/j.matbio.2024.01.002, PMID: 38219976

[19] Li Z Li Y Liu S Qin Z . Extracellular S100A4 as a key player in fibrotic diseases. J Cell Mol Med. (2020) 24:5973–83. doi: 10.1111/jcmm.15259, PMID: 32307910 PMC7294136

[20] Fei F Qu J Li C Wang X Li Y Zhang S . Role of metastasis-induced protein S100A4 in human non-tumor pathophysiologies. Cell Biosci. (2017) 7:64. doi: 10.1186/s13578-017-0191-1, PMID: 29204268 PMC5702147

[21] Li ZH Dulyaninova NG House RP Almo SC Bresnick AR . S100A4 regulates macrophage chemotaxis. Mol Biol Cell. (2010) 21:2598–610. doi: 10.1091/mbc.e09-07-0609, PMID: 20519440 PMC2912347

[22] Wong T Kang R Yun K . The multi-faceted immune modulatory role of S100A4 in cancer and chronic inflammatory disease. Front Immunol. (2025) 16:1525567. doi: 10.3389/fimmu.2025.1525567, PMID: 40078995 PMC11897520

[23] McHugh J . S100A4 inhibition targets fibrosis in SSc. Nat Rev Rheumatol. (2024) 20:67. doi: 10.1038/s41584-024-01080-1, PMID: 38212540

[24] Cui N Xu X Zhou F . Single-cell technologies in psoriasis. Clin Immunol. (2024) 264:110242. doi: 10.1016/j.clim.2024.110242, PMID: 38750947

[25] Do TH Ward NL Gudjonsson JE . Understanding psoriatic disease at single-cell resolution: an update. Curr Opin Rheumatol. (2025) 37:254–60. doi: 10.1097/BOR.0000000000001085, PMID: 40160177

[26] Qie C Jiang J Liu W Hu X Chen W Xie X . Single-cell RNA-Seq reveals the transcriptional landscape and heterogeneity of skin macrophages in Vsir(-/-) murine psoriasis. Theranostics. (2020) 10:10483–97. doi: 10.7150/thno.45614, PMID: 32929361 PMC7482809

[27] Wang J Li X Zhang P Yang T Liu N Qin L . CHRNA5 is overexpressed in patients with psoriasis and promotes psoriasis-like inflammation in mouse models. J Invest Dermatol. (2022) 142:2978–87 e6. doi: 10.1016/j.jid.2022.04.014, PMID: 35513071

[28] Butler A Hoffman P Smibert P Papalexi E Satija R . Integrating single-cell transcriptomic data across different conditions, technologies, and species. Nat Biotechnol. (2018) 36:411–20. doi: 10.1038/nbt.4096, PMID: 29608179 PMC6700744

[29] Korsunsky I Millard N Fan J Slowikowski K Zhang F Wei K . Fast, sensitive and accurate integration of single-cell data with Harmony. Nat Methods. (2019) 16:1289–96. doi: 10.1038/s41592-019-0619-0, PMID: 31740819 PMC6884693

[30] Ianevski A Giri AK Aittokallio T . Fully-automated and ultra-fast cell-type identification using specific marker combinations from single-cell transcriptomic data. Nat Commun. (2022) 13:1246. doi: 10.1038/s41467-022-28803-w, PMID: 35273156 PMC8913782

[31] Yu G Wang LG Han Y He QY . clusterProfiler: an R package for comparing biological themes among gene clusters. OMICS. (2012) 16:284–7. doi: 10.1089/omi.2011.0118, PMID: 22455463 PMC3339379

[32] Qiu X Mao Q Tang Y Wang L Chawla R Pliner HA . Reversed graph embedding resolves complex single-cell trajectories. Nat Methods. (2017) 14:979–82. doi: 10.1038/nmeth.4402, PMID: 28825705 PMC5764547

[33] Aibar S Gonzalez-Blas CB Moerman T Huynh-Thu VA Imrichova H Hulselmans G . SCENIC: single-cell regulatory network inference and clustering. Nat Methods. (2017) 14:1083–6. doi: 10.1038/nmeth.4463, PMID: 28991892 PMC5937676

[34] Ritchie ME Phipson B Wu D Hu Y Law CW Shi W . limma powers differential expression analyses for RNA-sequencing and microarray studies. Nucleic Acids Res. (2015) 43:e47. doi: 10.1093/nar/gkv007, PMID: 25605792 PMC4402510

[35] Suo S Zhu Q Saadatpour A Fei L Guo G Yuan GC . Revealing the critical regulators of cell identity in the mouse cell atlas. Cell Rep. (2018) 25:1436–45 e3. doi: 10.1016/j.celrep.2018.10.045, PMID: 30404000 PMC6281296

[36] Shannon P Markiel A Ozier O Baliga NS Wang JT Ramage D . Cytoscape: a software environment for integrated models of biomolecular interaction networks. Genome Res. (2003) 13:2498–504. doi: 10.1101/gr.1239303, PMID: 14597658 PMC403769

[37] Jin S Guerrero-Juarez CF Zhang L Chang I Ramos R Kuan CH . Inference and analysis of cell-cell communication using CellChat. Nat Commun. (2021) 12:1088. doi: 10.1038/s41467-021-21246-9, PMID: 33597522 PMC7889871

[38] Joost S Zeisel A Jacob T Sun X La Manno G Lonnerberg P . Single-cell transcriptomics reveals that differentiation and spatial signatures shape epidermal and hair follicle heterogeneity. Cell Syst. (2016) 3:221–37 e9. doi: 10.1016/j.cels.2016.08.010, PMID: 27641957 PMC5052454

[39] Yin X Yang Z Zhu M Chen C Huang S Li X . ILF2 contributes to hyperproliferation of keratinocytes and skin inflammation in a KLHDC7B-DT-dependent manner in psoriasis. Front Genet. (2022) 13:890624. doi: 10.3389/fgene.2022.890624, PMID: 35586566 PMC9110045

[40] Hu A Pickup ME Lawal MA Patel HJ Ahmed MI . The involvement of Elf5 in regulating keratinocyte proliferation and differentiation processes in skin. PloS One. (2025) 20:e0316134. doi: 10.1371/journal.pone.0316134, PMID: 39752333 PMC11698348

[41] Zhu WJ Li P Wang L Xu YC . Hypoxia-inducible factor-1: A potential pharmacological target to manage psoriasis. Int Immunopharmacol. (2020) 86:106689. doi: 10.1016/j.intimp.2020.106689, PMID: 32585606

[42] Sporl F Korge S Jurchott K Wunderskirchner M Schellenberg K Heins S . Kruppel-like factor 9 is a circadian transcription factor in human epidermis that controls proliferation of keratinocytes. Proc Natl Acad Sci U S A. (2012) 109:10903–8. doi: 10.1073/pnas.1118641109, PMID: 22711835 PMC3390879

[43] Lopez-Molina L Conquet F Dubois-Dauphin M Schibler U . The DBP gene is expressed according to a circadian rhythm in the suprachiasmatic nucleus and influences circadian behavior. EMBO J. (1997) 16:6762–71. doi: 10.1093/emboj/16.22.6762, PMID: 9362490 PMC1170280

[44] Gangwar RS Gudjonsson JE Ward NL . Mouse models of psoriasis: A comprehensive review. J Invest Dermatol. (2022) 142:884–97. doi: 10.1016/j.jid.2021.06.019, PMID: 34953514 PMC10190156

[45] Vicic M Kastelan M Brajac I Sotosek V Massari LP . Current concepts of psoriasis immunopathogenesis. Int J Mol Sci. (2021) 22:11574. doi: 10.3390/ijms222111574, PMID: 34769005 PMC8584028

[46] Babaie F Omraninava M Gorabi AM Khosrojerdi A Aslani S Yazdchi A . Etiopathogenesis of psoriasis from genetic perspective: an updated review. Curr Genomics. (2022) 23:163–74. doi: 10.2174/1389202923666220527111037, PMID: 36777004 PMC9878828

[47] Holbrook J Lara-Reyna S Jarosz-Griffiths H McDermott M . Tumour necrosis factor signalling in health and disease. F1000Res. (2019) 8:F1000. doi: 10.12688/f1000research.17023.1, PMID: 30755793 PMC6352924

[48] Preedy MK White MRH Tergaonkar V . Cellular heterogeneity in TNF/TNFR1 signalling: live cell imaging of cell fate decisions in single cells. Cell Death Dis. (2024) 15:202. doi: 10.1038/s41419-024-06559-z, PMID: 38467621 PMC10928192

[49] Paramio JM Segrelles C Casanova ML Jorcano JL . Opposite functions for E2F1 and E2F4 in human epidermal keratinocyte differentiation. J Biol Chem. (2000) 275:41219–26. doi: 10.1074/jbc.M004973200, PMID: 11005809

[50] Li Y Pi XY Boland K Lad S Johnson K Verfaillie C . Hmga2 translocation induced in skin tumorigenesis. Oncotarget. (2017) 8:30019–29. doi: 10.18632/oncotarget.16272, PMID: 28415789 PMC5444722

[51] Boudjelal M Wang Z Voorhees JJ Fisher GJ . Ubiquitin/proteasome pathway regulates levels of retinoic acid receptor gamma and retinoid X receptor alpha in human keratinocytes. Cancer Res. (2000) 60:2247–52., PMID: 10786691

[52] Inoue K Aoi N Sato T Yamauchi Y Suga H Eto H . Differential expression of stem-cell-associated markers in human hair follicle epithelial cells. Lab Invest. (2009) 89:844–56. doi: 10.1038/labinvest.2009.48, PMID: 19506554

[53] Tommasi C Yogev O Yee MB Drousioti A Jones M Ring A . Upregulation of keratin 15 is required for varicella-zoster virus replication in keratinocytes and is attenuated in the live attenuated vOka vaccine strain. Virol J. (2024) 21:253. doi: 10.1186/s12985-024-02514-8, PMID: 39385182 PMC11465976

[54] Bose A Teh MT Mackenzie IC Waseem A . Keratin k15 as a biomarker of epidermal stem cells. Int J Mol Sci. (2013) 14:19385–98. doi: 10.3390/ijms141019385, PMID: 24071939 PMC3821562

[55] Langlois S Maher AC Manias JL Shao Q Kidder GM Laird DW . Connexin levels regulate keratinocyte differentiation in the epidermis. J Biol Chem. (2007) 282:30171–80. doi: 10.1074/jbc.M703623200, PMID: 17693411

[56] Zhang XF Cui X . Connexin 43: Key roles in the skin. BioMed Rep. (2017) 6:605–11. doi: 10.3892/br.2017.903, PMID: 28584630 PMC5449964

[57] O'Shaughnessy EM Duffy W Garcia-Vega L Hussey K Burden AD Zamiri M . Dysregulation of connexin expression plays a pivotal role in psoriasis. Int J Mol Sci. (2021) 22:6060. doi: 10.3390/ijms22116060, PMID: 34199748 PMC8200029

