# Supplementary material for: Single-cell transcriptomics reveals keratinocyte dynamic processes associated with S100a4 expression in psoriasiform dermatitis
Source: Front Immunol. 2026 Jan 23;16:1744860. doi: 10.3389/fimmu.2025.1744860 (PMC12876221; doi:10.3389/fimmu.2025.1744860)
Supplement: Supplementary file 1 [file Table1.docx]

Supplementary Material

**Supplemental Figures**


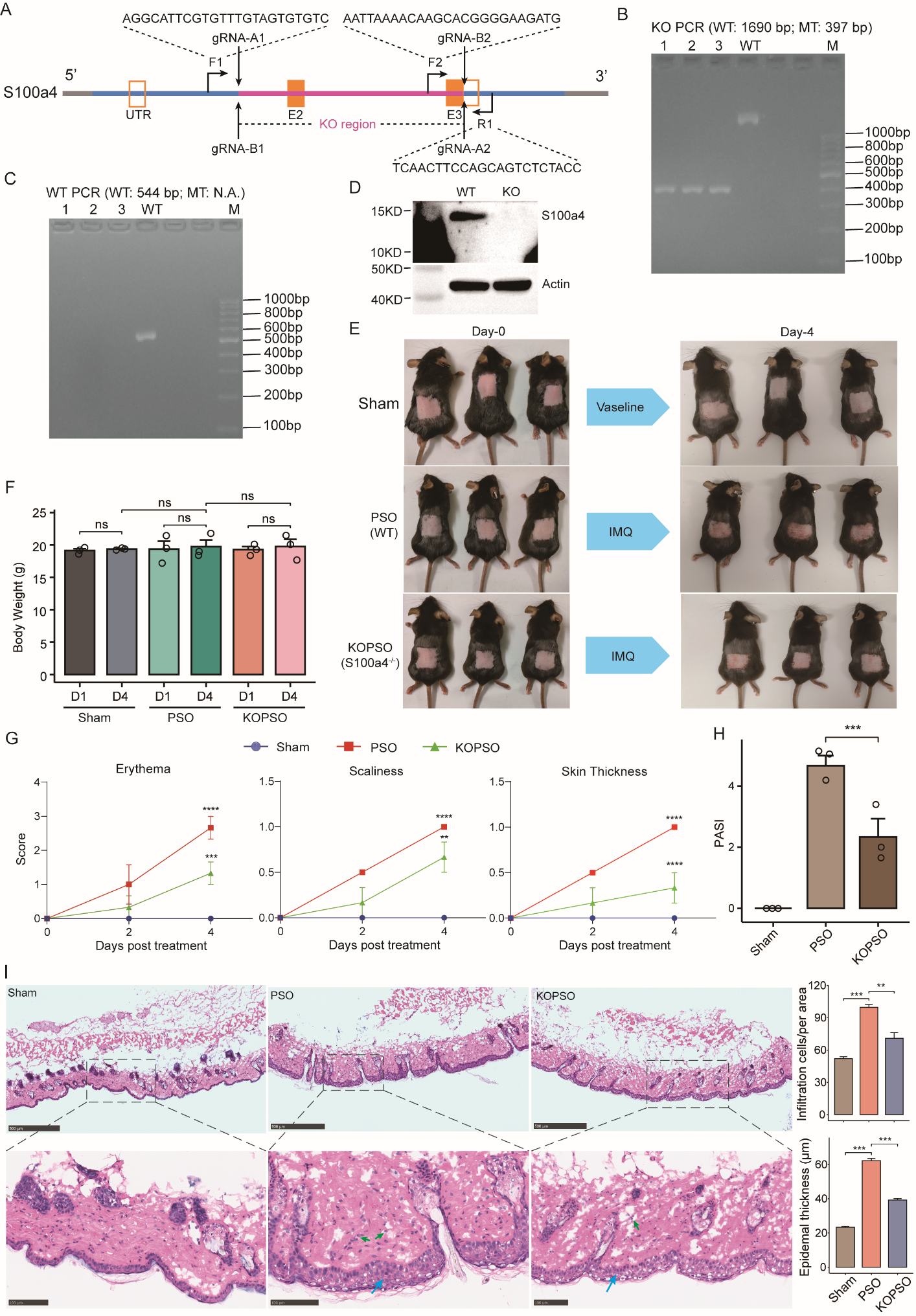


Supplementary Figure 1. S100a4^-/-^ effectively improves skin lesions in psoriasis mice. (A) Schematic diagram of CRISPR-Cas9 gene editing to create the S100a4^-/-^ mice. For global knockout, two gRNAs (A1 and A2) were used to generate a deletion, resulting in the S100a4 knockout allele. For genotyping PCR, the primer pair F1/R1 (flanking the deleted region) yields a specific to the knockout allele, whereas the F2/R1 amplifies the wild‑type allele. The approximate locations of the gRNA target sites and primers are indicated in the diagram. (B) Genotyping PCR products of mutant S100a4 transcripts were identified positive in mice homozygous for the KO allele. (C) Genotyping PCR products of wild-type S100a4 transcripts were identified negative. WT: wild type; MT: mutant type; N.A.: not applicable; M: marker. (D) Western blot confirms absence of S100a4 protein in the skin of KO mice. Actin serves as a loading control. (E) Schematic diagram of mouse treatment used in this study. IMQ: imiquimod. (F) Comparative analysis of body weight in mice. ns: not significant. (G) Comparative analysis of erythema, scaliness and skin thickness scores in mice (PSO vs. Sham and KOPSO vs. PSO). (H) Comparative analysis of PASI scores in mice. (I) H&E staining on skin sections from Sham, PSO and KOPSO mice. Scale bars correspond to 500 μm and 100 μm. Blue and green arrows indicate epidermal thickness and inflammatory infiltration, respectively. **p < 0.01, ***p < 0.001, and ****p < 0.0001.


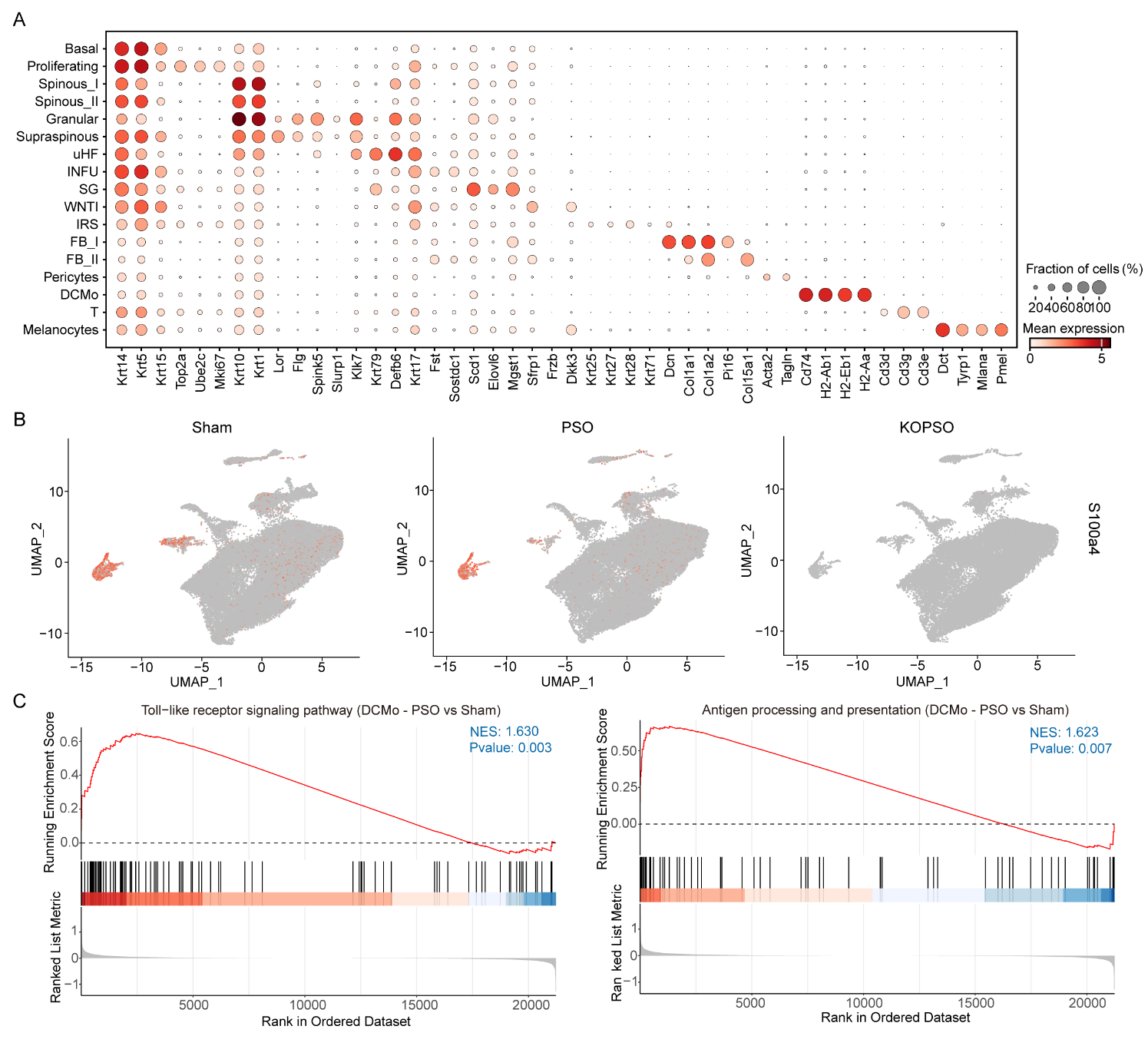


Supplementary Figure 2. Identification of relevant cell types and functional properties in psoriasis. (A) Dotplot showing expression of representative markers in each cell type. (B) UMAP plot showing the expression distribution of S100a4 in each group. (C) GSEA of “Toll−like receptor signaling pathway” and “Antigen processing and presentation” in DCMo based on PSO vs Sham.


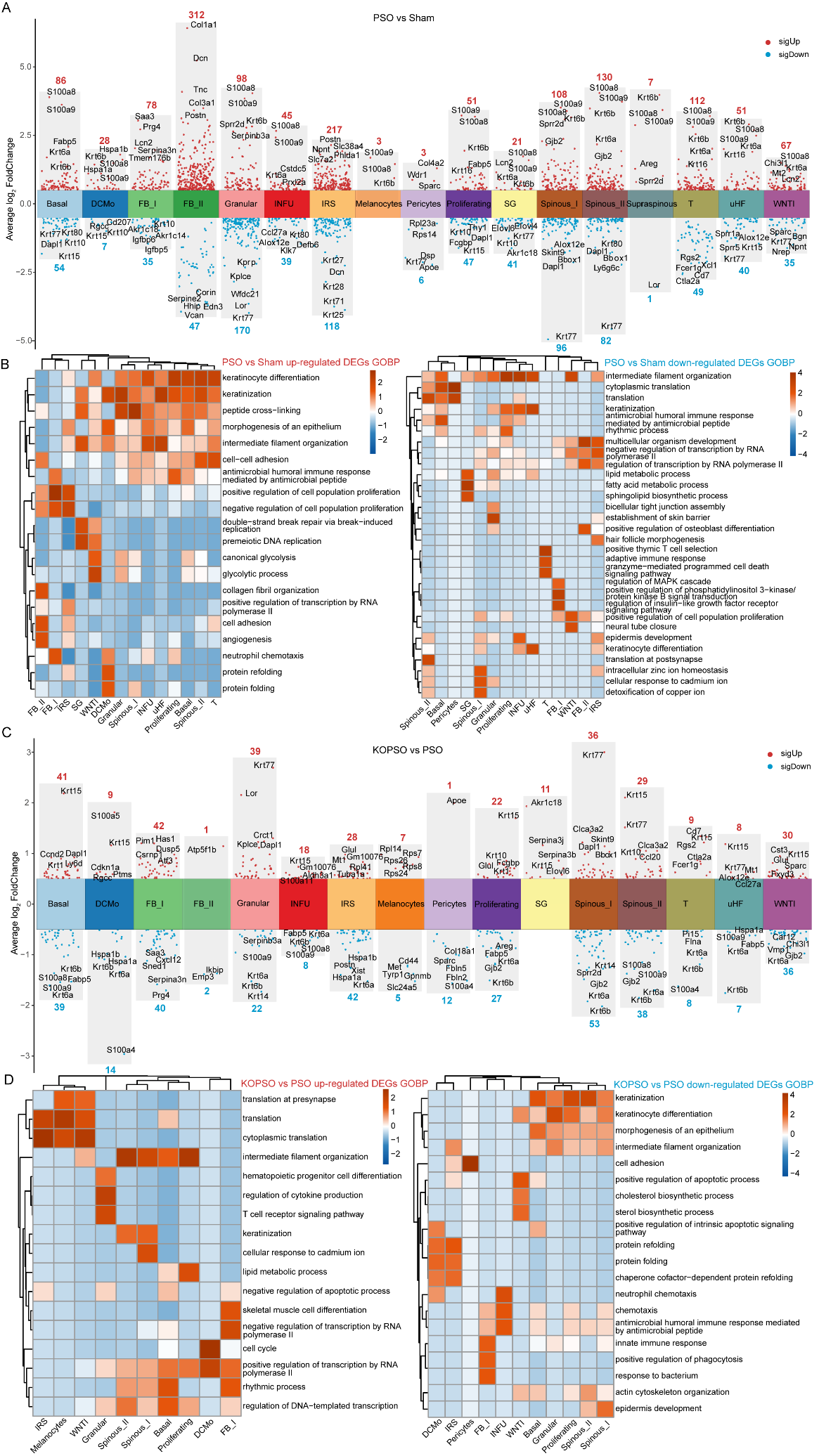


**Supplementary Figure 3.** Differentially expressed gene profiles and functional enrichment pathways across distinct cell types. **(A)** Volcano plot showing DEGs between the PSO and Sham groups in each cell type. Red indicates up-regulated genes; blue indicates down-regulated genes. Numbers denote gene counts. **(B)** Left and right panels display the top enriched GO biological processes for up-regulated and down-regulated DEGs, respectively, in the PSO vs Sham comparison across cell types. **(C)** Volcano plot showing DEGs between the KOPSO and PSO groups in each cell type. **(D)** Left and right panels display the top enriched GO biological processes for up-regulated and down-regulated DEGs, respectively, in the KOPSO vs PSO comparison across cell types.


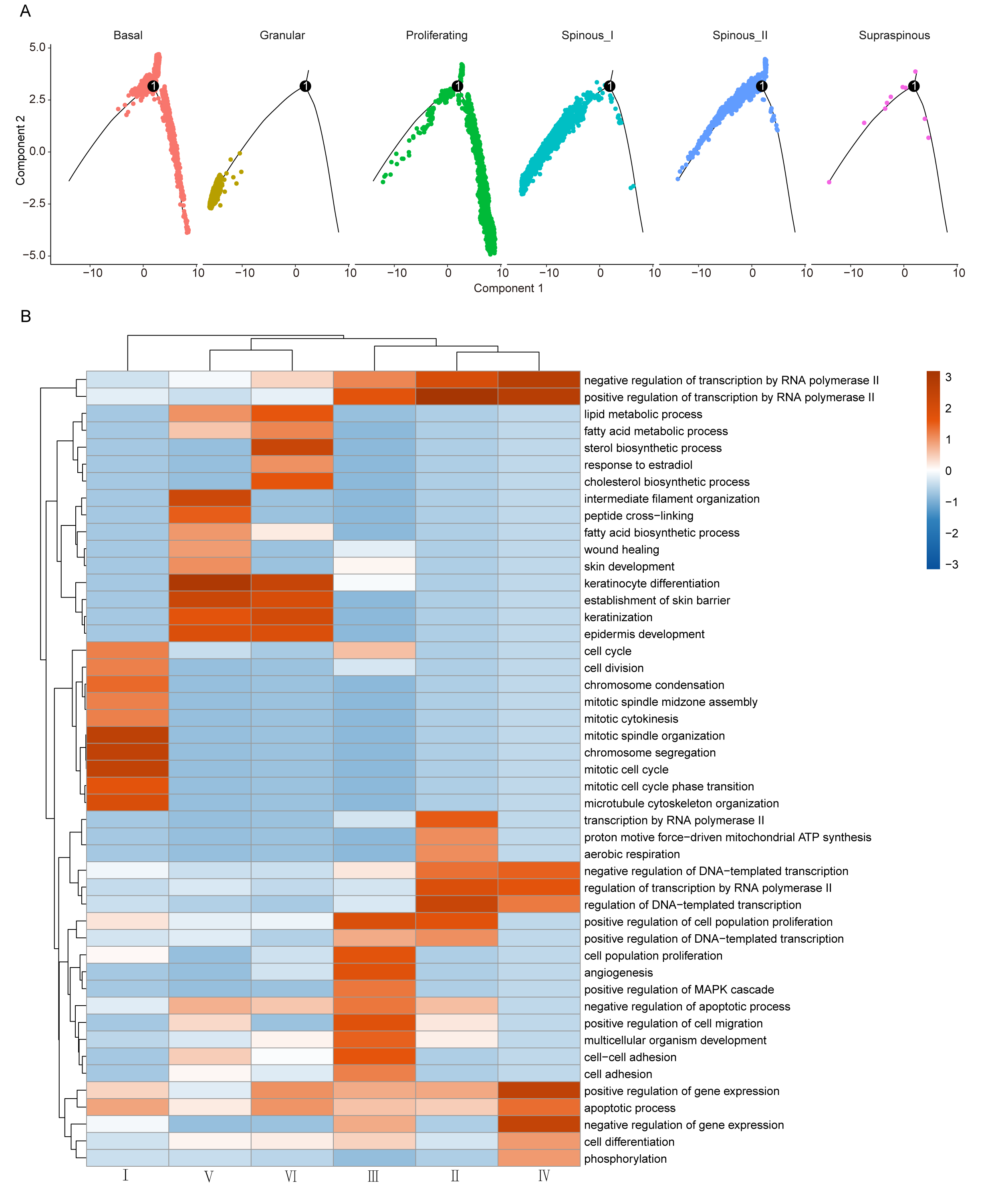


**Supplementary Figure 4.** Pseudotemporal trajectories and functional expression patterns of KCs. **(A)** Pseudo-time trajectories distribution of different KCs. **(B)** GO biological process results of different pseudo-temporal gene expression patterns. Top 10 terms were selected for each pattern and heatmap shows the enrichment q-value of these terms (scaled by column).


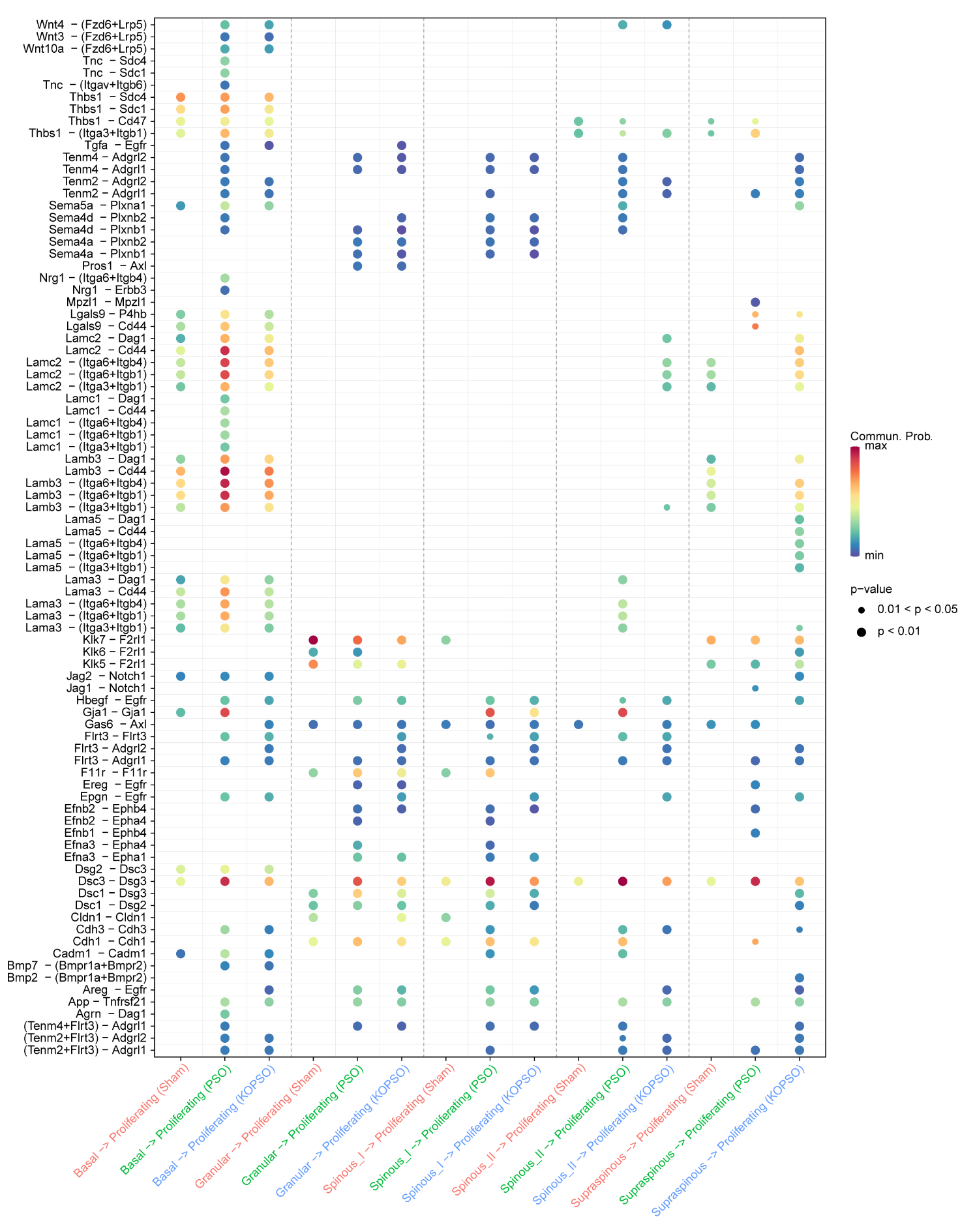


**Supplementary Figure 5.** Differential signaling pathways from other KC types to proliferating KCs.
